# A unified framework for the integration of multiple hierarchical clusterings or networks from multi-source data

**DOI:** 10.1186/s12859-021-04303-4

**Published:** 2021-08-04

**Authors:** Audrey Hulot, Denis Laloë, Florence Jaffrézic

**Affiliations:** 1 Université Paris-Saclay, INRAE, AgroParisTech, GABI , 78350 Jouy-en-Josas, France; 2 Université Paris-Saclay, AgroParisTech, INRAE, UMR MIA-Paris , 75005 Paris, France; 3 Université Paris-Saclay, UVSQ, Inserm, Infection et inflammation , 78180 Montigny-le-Bretonneux, France

**Keywords:** Data integration, Clustering, Network, MDS, MFA

## Abstract

**Background:**

Integrating data from different sources is a recurring question in computational biology. Much effort has been devoted to the integration of data sets of the same type, typically multiple numerical data tables. However, data types are generally heterogeneous: it is a common place to gather data in the form of trees, networks or factorial maps, as these representations all have an appealing visual interpretation that helps to study grouping patterns and interactions between entities. The question we aim to answer in this paper is that of the integration of such representations.

**Results:**

To this end, we provide a simple procedure to compare data with various types, in particular trees or networks, that relies essentially on two steps: the first step projects the representations into a common coordinate system; the second step then uses a multi-table integration approach to compare the projected data. We rely on efficient and well-known methodologies for each step: the projection step is achieved by retrieving a distance matrix for each representation form and then applying multidimensional scaling to provide a new set of coordinates from all the pairwise distances. The integration step is then achieved by applying a multiple factor analysis to the multiple tables of the new coordinates. This procedure provides tools to integrate and compare data available, for instance, as tree or network structures. Our approach is complementary to kernel methods, traditionally used to answer the same question.

**Conclusion:**

Our approach is evaluated on simulation and used to analyze two real-world data sets: first, we compare several clusterings for different cell-types obtained from a transcriptomics single-cell data set in mouse embryos; second, we use our procedure to aggregate a multi-table data set from the TCGA breast cancer database, in order to compare several protein networks inferred for different breast cancer subtypes.

**Supplementary Information:**

The online version contains supplementary material available at 10.1186/s12859-021-04303-4.

## Background

When integrating data in computational biology, we are often confronted with the problem of comparing outcomes from different types of data, with various forms of representations [1–3]. These representations may either result from a learning algorithm (e.g. dimension reduction, hierarchical clustering or network inference) or they may be extracted from a data base, reflecting our knowledge about a complex biological process.

As a simple example in genomics, several hierarchical clusterings of individuals can be obtained based on transcriptomics, proteomics or metagenomics experiments, giving birth to several tree-like representations which need to be compared and eventually aggregated. Such an analysis is essential to better understand the data and to obtain a consensus clustering from coherent trees.

A review of omics data integration methods is provided by Ritchie et al. [4] in a prediction perspective, which also applies to exploratory and unsupervised questions like clustering. In [4], data integration methods are classified into three categories: concatenation-based integration, transformation-based integration and model-based integration. For the last two categories, different omics and different types of objects can be integrated together in theory. However, most methods developed for this purpose and described in [4] involve similar objects for integration in practice.

Among them, a majority consider that the original data tables, i.e. data in the form of a table with observations and features, from which the objects are derived are available, which is not always the case in reality.

Regarding objects provided in the form of trees or networks, the literature is more specific, and treats separately the question of comparing such objects or of creating consensus from a collection of them. A detailed review is given in [5] on the question of network comparison, which usually involves a representation of those networks by the adjacency matrices or using methods for a graph embedding [6]. Comparison of a set of trees often relies on distances between trees, for example using Robinson-Foulds metric [7, 8] as in phylogenetics. Creating a consensus out of a set of objects is a natural next step in the integration process following the comparison of objects, hence it is a recurring question in research area studying data integration.

The procedure that we introduce in this paper answers the comparison and integration questions simultaneously, and can be applied to a variety of data representation broader than just tree or network structures. In a nutshell, the contribution of this paper is a unified and simple way of comparing and integrating data with various representation forms (like trees, networks or factorial maps). It relies on a two-step strategy which philosophy is close to unsupervised multiple kernels: the first step consists in finding a way to project all these objects into a comparable coordinate system.

This leads to new collection of data tables which are analyzed in a second step by means of any multi-table integration method. The specificity of our approach is to combine multidimensional scaling (MDS) [9, 10] and Multiple Factor Analysis (MFA) [11–13] to perform these two steps: the MDS allows us to calculate coordinates from distances or dissimilarities, obtained from trees, networks or factorial maps. Then, MFA provides a canonical framework to perform multi-table analysis, bringing powerful tools to study the relationships between tables of data, and to quantify the similarities and differences between them. In fact, our process fits into the multiple kernel methods framework [2, 14, 15]. We define here a procedure where everything is automated for the integration process, as the user has very few, if none, parameters to define.

Our procedure is particularly useful in the case where we are given a set of trees or networks, or any object set we want to compare, without the original data. For example, networks of protein-protein interaction or ecological networks are available on databases without any indication of the data they have been built on [16–18]. This can also be useful to compare different ways to transform the data, e.g. using different distances or aggregation criteria to build the trees.

The rest of the paper is organized as follows: first, we give details about the proposed methodology. Then its performance is evaluated on simulated data and compared to a multiple kernel integration method, and two real-world data sets are analyzed: the first one is a single-cell data set that illustrates the comparison of clusterings for different cell-types in mouse embryos. The second one is a -omic data set from the TCGA breast cancer database, for which several PPI networks are compared and aggregated for different breast cancer subtypes.

## Methods

**Fig. 1:**
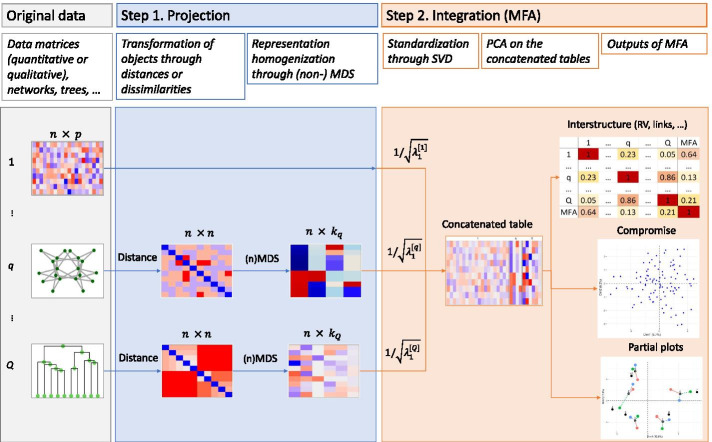
Workflow description of the process

In order to compare and aggregate trees or networks, in the context of multi-source data analysis, we adopt the general 2-step approach described in Fig. 1, which can be summarized as follows:

1. **Projection.** This step aims at projecting every source of data that are not available in the form of data tables, i.e. in a form with observations and features, into a Euclidean space. This is achieved as follows:
  - Represent all data sources other than data tables in the form of distance or dissimilarity matrices.
  - Place these distances in the same coordinate system.
2. **Integration.** In this step, all data tables available are integrated together.
  - Apply multi-table analysis This integration is achieved through a multitable method, e.g MFA, multiple coinertia analysis [19], STATIS [20, 21], multiblock PLS [22]…, see [23] for a recent review of multitable integration methods.
  - Use factorial representation for comparing the projected data and creating a consensus.

Step 1 is done by retrieving a distance matrix specific to either trees or networks (see details below) and then applying multidimensional scaling (MDS), which provides a new set of coordinates from all these pairwise distances. These new coordinates can be interpreted the same way as original multi-source data and all methods available for the analyses of such data sets can be used for Step 2 (integration). We chose Multiple Factor Analysis (MFA), which allows us to position the different objects on a factorial map. These two methods require to chose the number of axes to retain.

Any object that can be summarised in the form of a dissimilarity matrix can be integrated using the two steps. We would like to point out that any data, categorical or quantitative (original data, factorial maps, clinical outcome…), as long as it is computed on the same individuals, can be integrated in step two.

This provides tools for identifying objects that have similar patterns across various conditions, for positioning them on maps and for creating groups of objects that are interesting to aggregate together. Once the groups of objects are formed, MFA axes allow to create further analyses at the individual level, such as consensus hierarchical clustering.

### Multidimensional scaling

We will refer here to the classical Multidimensional scaling (MDS), introduced by [9]. The goal of the method is to find coordinates *X* of data given a dissimilarity matrix $\Delta$ between individuals.

Consider a matrix of dissimilarities $\Delta$, and $\Delta ^2$ the matrix of squared coefficients of $\Delta$, the double-centered matrix is defined as $B = -\frac{1}{2}J\Delta ^2J$, where $J = I - \frac{1}{n} {\mathbbm{1}} {\mathbbm{1}}^T$ is the centering matrix. The classical scaling [10, 24] minimizes the strain: $\left\Vert XX^T - B\right\Vert ^2$ where *X* are the coordinates we search for. The solution can be shown to verify $X = Q_{+}\Lambda _{+}^{1/2}$ with $\Lambda _{+}$ being the diagonal matrix with the non negative and non-zero eigenvalues of *B*, and $Q_{+}$ the corresponding eigenvectors. If $\Delta$ is a Euclidean distance matrix, which according to [25, 26] is equivalent to $-\frac{1}{2}J\Delta ^2J$ being positive semi-definite, the MDS coordinates *X* are actually the original coordinates up to a rotation and a translation if *X* is not column-centered (thus equivalent to Principal Component Analysis). In a context of Euclidean dissimilarity matrix, MDS is also equivalent to kernel-PCA [27, 28]. Indeed, *B* is a positive-definite matrix and hence a kernel.

Several variants of MDS exist to deal with matrices that are not positive semi-definite, such as the Cailliez’ method [29], which consists in adding a positive constant to the element outside of the diagonal to make the matrix positive definite. [30] proposed a similar method by adding a constant to the squared dissimilarities and taking the square root as the modified distances. When the dissimilarities are not produced by a distance function (metric), solutions for non-metric MDS are also available [31, 32]. In all our applications, we chose to take only the positive eigenvalues of *B* when needed.

Our process yields the $X = Q_{+}\Lambda _{+}^{1/2}$ matrix for each object in the first step, and passes them to the second step, the Multiple Factor Analysis, along with additional data tables if any are available.

### Multiple factor analysis

Multiple Factor Analysis (MFA) is a method to jointly analyze several possibly heterogeneous data sets  [11, 12]. Let $X_1, \ldots , X_Q$ be *Q* data tables, which can be either quantitative or qualitative data, with $p_1, \ldots , p_Q$ features observed for the same *n* individuals. In the context of this paper, some if not all of the $X_q$ are provided by the first step of the process: the MDS.

The principle of MFA is to divide each data table by its first singular value to ensure the contributions of the data sets in the first axis are equal. Data tables are then concatenated and a PCA is performed on the concatenation of $X_1, \ldots , X_Q$ each divided by its first singular value. This step is called global PCA in [12]. We will refer to it as gPCA in the following.

In the context of Euclidean distances, the first step of the MFA is redundant with the MDS and a unique MFA can be performed on the double centered distance matrices *B* as input. As in this case *B* is a kernel matrix, using MFA on a set of *B* matrices can also be seen as a kernel-MFA, given the equivalence of kernel-PCA and MDS. The combination of MDS and MFA can be considered as an extension of the MFA to the non-Euclidean dissimilarities.

A great advantage of the use of MFA in integrating data is that it provides several scores to compare the different tables, as well as axis coordinates that allow the visualization of features, individuals and tables on a factorial map. In this study, we will use in particular the group coordinates obtained from the MFA analysis.

#### Group coordinates

The data sets $X_1, \ldots , X_Q$ can be positioned on each component using their contribution to the gPCA . Let $\tilde{X}$ be the concatenation of $X_1, \ldots , X_Q$ each divided by its first singular value. The gPCA factorizes $\tilde{X}$ with singular value decomposition into $U \Lambda V^T$, where *V* is the matrix of the loadings. The loadings can be decomposed into subsets $V = [V_{(1)}, \ldots , V_{(Q)}]$ delimited by the number of variables in each table. With $\lambda _\ell$ the $\ell$th entry of $\Lambda$, the coordinate of table $X_q$ along axis $\ell$ is defined by

1 $$\begin{aligned} {\text{coord}}_{q,\ell } = \lambda _\ell \times \sum _{j = 1}^{p_q} V_{(q)\,\ell ,j}^2 = \lambda _\ell \times {\text{ctrb}}_{q, \ell }, \end{aligned}$$

with $p_q$ being the number of variables of table $X_q$, and ${\text {ctrb}}_{q, \ell }$ the contribution of table *q* on dimension $\ell$ of the gPCA.

Using these group coordinates, we propose to create a clustering of the tables. In the following, we use hierarchical clustering, but any clustering method can be considered. The tables are then gathered according to their similarity and can be analyzed together within groups.

### Creating a consensus from MFA results

In this section we give details about how we intend to build consensus trees and networks from MFA results, as it is the multi-table analysis chosen here. As mentioned earlier, other multi-table methods could also be used.

To compute a consensus hierarchical clustering given the MFA results, we will refer to the clusters made on the group coordinates. Let ${\mathcal {T}}_1, \ldots , {\mathcal {T}}_{k_1}$ be a group of trees defined as previously described. The same process of cophenetic distances, MDS and MFA is applied on these trees. A consensus clustering is then obtained by performing a hierarchical clustering (or any other clustering method) on the individual coordinates obtained by the MFA.

When creating a network consensus, once the groups of networks are formed using the group coordinates of the MFA, a consensus network is created by using a majority rule consensus on the original adjacency matrices, i.e. an edge is kept if it is present in more than half of the networks in the identified groups.

## A common representation for trees and networks

This section details the different ingredients used in the method presented above: we explain how the distance matrices can be retrieved when focusing on network or tree structures, although any object that can be represented by a distance or dissimilarity matrix can be used in our procedure.

### Retrieve a distance matrix from a tree

Consider a hierarchical tree obtained with any hierarchical clustering (it can be a non-binary tree). Recall that the cophenetic distance between two leaves of a tree is the height where the two leaves or their cluster are merged. Hierarchical trees can then be summarized by a symmetrical matrix using the cophenetic distance [33]. In the context of MDS, it is best to use Euclidean distances to avoid numerical issues while computing the coordinates. It is shown in [34] that the distances extracted from a ultrametric tree can always be considered as Euclidean distances. All hierarchical clusterings built on a distance and aggregation criterion are ultrametric trees, therefore applying MDS to a cophenetic matrix requires no further transformation of the matrix in this particular case.

### Retrieve a distance matrix from a network

Consider an undirected binary graph: we suggest to build a distance matrix from this graph by means of the shortest path distance between all pairs of nodes, before applying MDS. The shortest path distance is defined as the minimum number of edges to cross to go from one node to another. The shortest path distance between two unconnected nodes is generally set to infinity. This method can also be applied to weighted graphs with positive weights, where the cost of a path is understood as the sum of weights along the edges of the path.

## Results

In this section we describe the results obtained on simulated data, in order to evaluate the performances of the proposed method, as well as on two real data sets. Analyses were performed with R 4.0.2 [35]. All code and data are available at https://github.com/AudreH/intTreeNet.

Hierarchical clustering was performed using Euclidean distance and Ward’s aggregation criterion as implemented in the “ward.D2” option of the *hclust* R function [36]. All trees were transformed using the *cophenetic* base function. Using *cmdscale*, the new data coordinates from the MDS approach were obtained. MFA was performed using the MFA function from the factoMineR package [37]. To assess the differences between clusterings, we used the *Adjusted Rand Index* (ARI) [38, 39] from the aricode R-package [40], which measures the agreement between two classifications. To determine the groups in a hierarchical clustering, we used the DynamicTreeCut method as implemented in the R-package of the same name [41]. This method identifies groups based on the structure of the tree and the distance matrix used to build the tree. In the graph application, the shortest path distance is computed using the *distances* function of the igraph R-package [42] and default parameters.

We compare the results of our process to the ones obtained by combining kernels. When needed, distance or dissimilarity matrices $\Delta$ are transformed into similarities using the double-centering formula: $B = -\frac{1}{2}J\Delta ^2J$. To ensure that matrix *B* can be considered a kernel, it is reconstructed using only positive eigenvalues. For the inverse transformation, from a similarity (kernel) matrix *S* to a distance/dissimilarity one, we use the formula: $\forall (i,i'),~ \Delta _{ii'} = \sqrt{S_{ii} + S_{i'i'} -2S_{ii'}}$.

To compare the kernels between them, we use the similarity coefficient computed as the cosine of the Frobenius norm between kernel matrices, as described in [2]. The matrix of these coefficients is then transformed into dissimilarity, and hierarchical clustering is performed using complete-linkage.

We use the mixKernels R-package [2] with the option “full-UMKL” (full Unsupervised Multiple Kernel Learning) and default parameters to find a consensus kernel after we identify the kernel clusters.

### Simulation study in the case of clusterings

In this first set of simulations, $Q = 9$ tables with $p = 1000$ variables and $n = 100$ individuals were generated according to three different patterns of classification with $K = 4, 3$ and 5 groups for each pattern, respectively. The chosen patterns of classifications are very different, with an ARI close to 0 between them. Observation *j* for individual *i* of table *q* when *i* is in group *k* follows a Gaussian distribution, i.e.,

2 $$\begin{aligned}&i\in \{1,\ldots ,n\}, \,\,\, j\in \{1,\ldots ,p\}, \,\,\, k \in \{1,\ldots ,K_q\} , \,\, q \in \{1,\ldots ,Q\}, \nonumber \\&\qquad i\in k , \,\,\,\, Y_{i,j}^q = {\mathcal {N}}(\mu _k, q^2) \end{aligned}$$

Each observation is generated according to Eq. (2), with the mean depending on the group of the individual and the variance depending on the table number. A total of 9 trees of 100 individuals were built from these tables and MDS was performed on each cophenetic distance matrix.

**Fig. 2:**
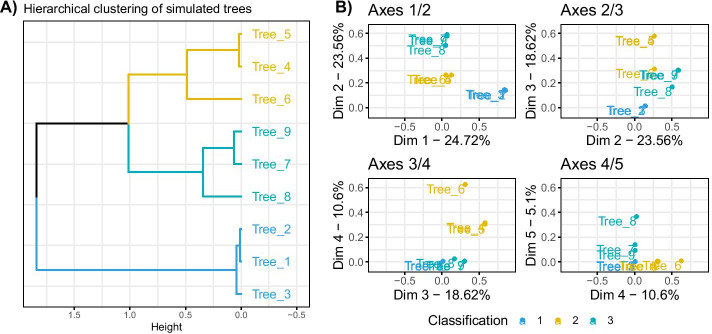
Results for the simulation study on hierarchical clustering data. 3 classifications with $K = 4, 3$ and 5 groups respectively were simulated following Eq. 2 (**A**) represents the hierarchical clustering obtained with the MFA group coordinates, performed with Euclidean distance and ward.D2 aggregation criterion. (**B**) represents the factorial map for axes 1 to 5 of the MFA, these group coordinates were used to compute the hierarchical clustering on (**A**)

Figure 2 presents the hierarchical clustering obtained on the coordinates of the trees and the factorial maps of the data set. Tables with the same classification are grouped together in the hierarchical clustering, as well as on the first two axes of the MFA. The first axis differentiates the tables from the first classification from the others, the second axis differentiates the tables from classification 3 from the rest. These observations made on the group coordinates are visible in the hierarchical clustering as the level of division between elements reflects the axis on which the separation is found (*e.g.* third division in the tree separates Tree 6 from its group, and is found on axis 3 of the MFA).

The hierarchical clustering performed on the group coordinates and the classification of the tables made by DynamicTreeCut can help identify the trees that are close in terms of underlying information. The three groups of trees that we identify using DynamicTreeCut are the three groups of tables we simulated.

This approach allowed to visualize and compare the different clusterings before calculating a consensus tree. In this example, it would not make sense to try to aggregate all the trees, as they have very different structures, given that the ARI between the classifications used to generate the data is close to 0, as mentioned above.

**Fig. 3:**
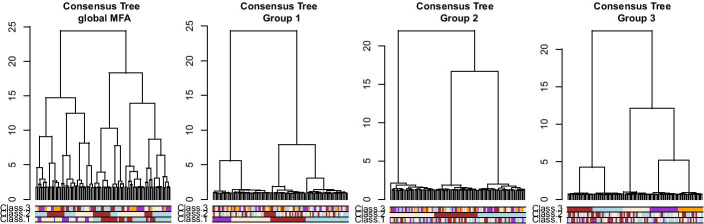
Results for the simulation study on hierarchical clustering data. Consensus trees obtained on 4 configurations, with colored bars representing the simulated classifications

The consensus trees can be obtained by performing a hierarchical clustering on the individual coordinates of the MFA axes (see Additional file 1). Results of the three consensus trees based on the identified sub-groups of data are presented in Fig. 3. As expected, inside a group of tables we retrieved the original classification, and did not find any information on the other classifications. On the other hand, in the consensus tree obtained with all the tables, none of the simulated classification patterns were recovered, with a maximum ARI of 0.51 obtained for classification 1 as shown in Table 1.

**Table 1:** **ARI results for the simulation study on hierarchical clustering**

	Global Consensus	Consensus Group 1	Consensus Group 2	Consensus Group 3
*MFA combination*				
Class. 1	**0.51**	**1**	0.01	0.04
Class. 2	0.34	0.02	**1**	0.03
Class. 3	0.22	0.01	0.02	**1**
*Kernel combination*				
Class. 1	0.24	**0.98**	0.01	0.04
Class. 2	0.25	0.01	**1**	0.01
Class. 3	**0.61**	0.01	0.01	**1**

Maximum ARI (*Adjusted Rand Index*) between each tree and simulated classification, for the MFA combination and kernel combination consensus trees. Bold font indicates the maximum ARI compared to the simulated classification, for each consensus tree

#### Comparison with kernel combination method

**Fig. 4:**
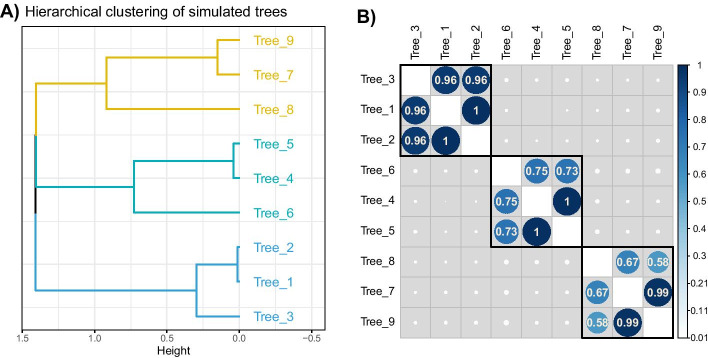
Results for the simulation study on hierarchical clustering data for kernel combination. 3 classifications with $K = 4, 3$ and 5 groups respectively were simulated following Eq. 2 (**A**) represents the hierarchical clustering obtained with the distance matrix derived from the consensus kernel, performed with complete linkage. **B** represents the C-coefficient between the cophenetic kernel tables, on which (**A**) was built and ordered according to the hierarchical clustering of (**A**)

**Fig. 5:**
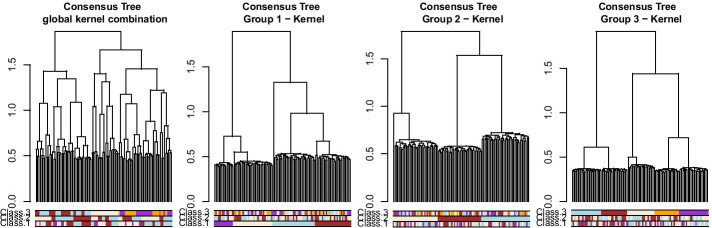
Results for the simulation study on hierarchical clustering data kernel combination. Consensus trees obtained on 4 configurations, with colored bars representing the simulated classifications

We transformed the cophenetic distances into similarities using the double centering formula. These new matrices are considered kernels as they are Gram matrices. The similarities between kernels are represented in Fig. 4. As for the previous results, there was a clear separation of the three groups of trees. The DynamicTreeCut package gave us 3 groups. The kernels corresponding to these groups were combined into 3 consensus kernels, then transformed into dissimilarity matrices. Hierarchical clustering with complete linkage was performed to retrieve the three corresponding consensus trees, as represented in Fig. 5, with the global consensus tree built on the global consensus kernel.

The three consensus trees, made on the three groups of trees, retrieve the simulated classification without difficulty in this situation. It is interesting to note that the two approaches give a tree with a similar grouping pattern, although the overall structures of these trees are slightly different. The consensus trees obtained on the global results for each method are also slightly different, as seen in Table 1: the global consensus made on MFA results is closer to the first classification while the global consensus of the kernel combination is closer to the third classification. This highlights the main difference between these approaches, i.e. the way of building the consensus either from the individual coordinates given by the MFA or with a combined kernel.

### Simulation study on network data

A similar simulation setup was used for the network data: $Q = 9$ adjacency matrices with $n = 100$ were simulated according to three different classification patterns, with an ARI close to 0 between them, of $K = 4, 3$ and 5 groups respectively. The presence or absence of an edge between two nodes was generated according to Eq. 3, with connection probabilities depending on the group the nodes are in. We chose $\pi _{kl} = 0.05$ for $k \ne l$ and $\pi _{kk} = 0.8$.

3 $$\begin{aligned}&i,j \in \{1,\ldots ,n\}, \,\,\, k,l \in \{1,\ldots ,K_q\} , \,\,\, q \in \{1,\ldots ,Q\}, \nonumber \\&\qquad i\in k ,\, j\in l \,\,\, A_{i,j}^q = {\mathcal {B}}(\pi _{kl}) \end{aligned}$$

**Fig. 6:**
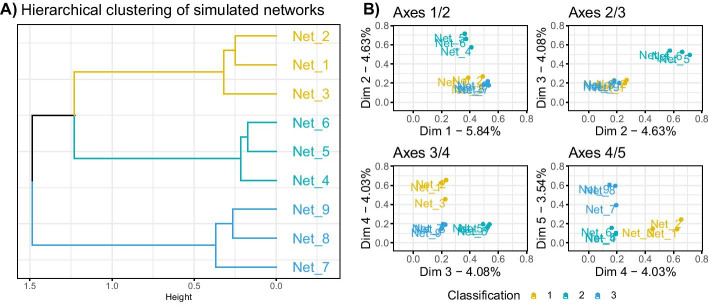
Results for the simulation study on network data. 3 classifications with $K = 4, 3$ and 5 groups respectively were simulated following Eq 3. **A** presents the hierarchical clustering of the networks based on the group coordinates of the MFA. **B** Shows the factorial maps for the 5 first axes of the MFA. These coordinates are the group coordinates on which (**A**) was made

The shortest path was then computed, and transformed into new data using the MDS. Results of the MFA are shown in Fig. 6, presenting the factorial maps for the objects, as well as a clustering obtained from the MFA coordinates.

**Fig. 7:**
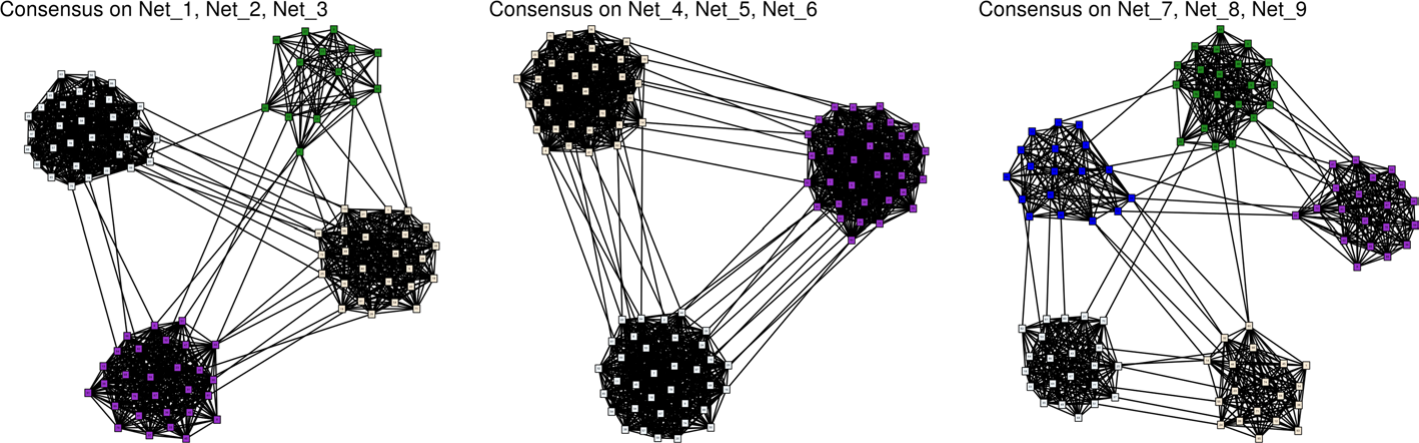
Simulation study on network data. Consensus network obtained on the networks clusters found with MFA. Nodes are colored according to their group used for simulating the data

Figure 7 shows the majority-vote consensus obtained with the groups formed by the hierarchical clustering. The original classifications were recovered very well in the networks, as the nodes are grouped in the network according to their simulated classification. The connection probability inside a cluster is far superior to the one between groups, which is exactly what we simulated. To provide a quantitative measure of the resemblance between the simulated networks and consensus obtained, we computed the true positive rate, false positive rate and the true discovery rate between the estimated and simulated networks, using the *compareGraphs* function of the pcalg R-package. The results are shown in Table 2. There is little difference between the consensus within each group and the simulated graphs, the true discovery rates are always greater than 0.8 between these networks.

**Table 2:** **Results on network simulations—comparison between consensus networks by groups found with MFA hierarchical clustering of networks**

	Net_1	Net_2	Net_3	Net_4	Net_5	Net_6	Net_7	Net_8	Net_9
*Consensus 1*									
tpr	0.85	0.84	0.84	0.27	0.27	0.26	0.30	0.29	0.29
fpr	0.05	0.05	0.05	0.24	0.24	0.24	0.24	0.24	0.24
tdr	0.85	0.86	0.86	0.33	0.33	0.32	0.24	0.24	0.23
*Consensus 2*									
tpr	0.32	0.33	0.32	0.87	0.87	0.87	0.33	0.32	0.33
fpr	0.30	0.30	0.30	0.06	0.06	0.06	0.30	0.30	0.30
tdr	0.26	0.27	0.26	0.85	0.86	0.86	0.21	0.21	0.21
*Consensus 3*									
tpr	0.22	0.23	0.22	0.20	0.21	0.20	0.79	0.80	0.81
fpr	0.18	0.17	0.18	0.18	0.18	0.18	0.04	0.03	0.03
tdr	0.29	0.31	0.30	0.33	0.34	0.33	0.84	0.86	0.85

The consensus networks found with Kernels combination results are identical

#### Comparison with kernel combination method.

**Fig. 8:**
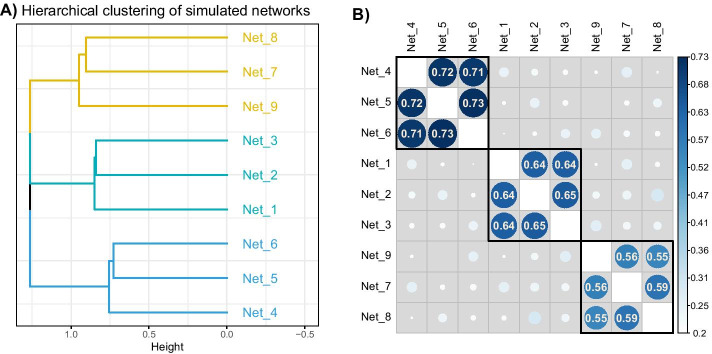
Results for the simulation study on network data with kernels combination. 3 classifications with $K = 4, 3$ and 5 groups respectively were simulated following Eq 3. **A** Represents the hierarchical clustering obtained with the distance matrix derived from the consensus kernel, performed with complete linkage. **B** Presents the C-coefficients of the network kernels, on which (**A**) was made

Using the same transformation on the shortest path distance matrices to find dissimilarities, we performed a kernel combination using mixKernels, and built a hierarchical clustering with complete linkage to find the tree and the similarities between kernels represented in Fig. 8. The tree gave us the same three groups as for the MFA results with a different junction structure, as already noticed in the tree simulations. The way of creating a consensus network for each of these groups does not change here: a majority vote applied on the adjacency matrices gave us the same results as the ones presented in Fig. 7.

### Application to single-cell data

In this application, we created hierarchical clusterings of genes given a set of data tables, and considered the trees as the only available source of data. We applied our procedure as well as the kernel approach to help group the trees together.

The data we used in this section are presented in [43]. They come from 411 mouse embryos, collected at different time points, from day 6.5 to day 8.5. Transcriptome expression is available for 116,312 cells. The authors divided these cells into 37 groups that we will call cell-types. For this application we only used the samples from the first stage (E6.5), deleted all genes that had a mean count of less than $10^{-3}$, as well as genes on the Y chromosome and the Xist gene, as the authors did in their analysis—the original code, and particularly the block of code that removes the Y chromosome and the Xist gene, can be found at https://github.com/MarioniLab/EmbryoTimecourse2018/blob/master/analysis_scripts/atlas/core_functions.R. These two steps led to the analysis of 15,086 genes and 3520 samples.

Following the procedure explained by [43], we selected the most variable genes using the scran R-package and the function *modelGeneVar*. In total, 318 genes were selected by taking a threshold of 0.1 for the adjusted p-values.

Samples were then divided according to their cell-type. Cell-types with only one sample were discarded. The cell-types and the number of samples for each one are presented in Table 3. This pre-processing of the data resulted in a set of 7 tables with transcriptome expression available for the same genes. One tree per table was then built, considering the genes as the leaves. We applied the method presented above on these trees in order to compare them, using the group coordinates of the MFA, and aggregate the most coherent ones. First, the MDS was applied to the trees from which 317 axes were obtained for each cell-type tree and used for the MFA analysis.

**Table 3:** **Number of samples per cell-type for the single cell application**

Group	Nb Samples
Epiblast	2276
ExE ectoderm	633
ExE endoderm	126
Nascent mesoderm	4
Parietal endoderm	10
Primitive Streak	381
Visceral endoderm	52

**Fig. 9:**
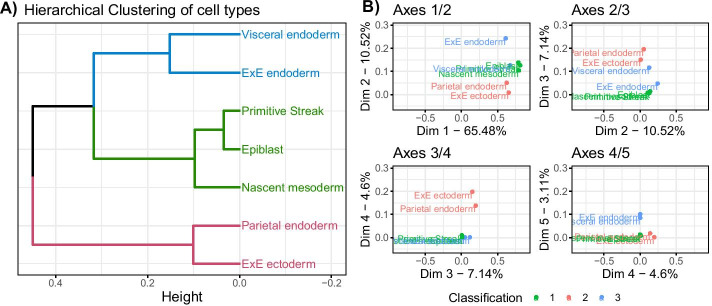
Visualization of groups given by MFA for the single-cell data application. (**A**) Dendrogram of the cell-types obtained on group coordinates of the MFA results using Euclidean distance and Ward’s aggregation criterion. Clusters were chosen using function *DynamicTreeCut* and colored accordingly. (**B**) Factorial maps for axes 1 to 5 of the MFA, these group coordinates were used to compute the hierarchical clustering on (**A**). Objects are colored according to their group in the tree

The cell-types were then grouped in clusters using a hierarchical clustering on their coordinates. Figure 9 shows this hierarchical clustering, as well as the factorial maps obtained with the MFA. Using the *DynamicTreeCut* function with minimal cluster size of 1, we defined three groups of cell-types.

In the supplementary data of [43], the authors presented a map of the cell-types for every timepoint. The map of E6.5 shows roughly three groups of cell-types: the first one consisting in Epiblast, Rostral neurectoderm, Primitive Streak, Surface ectoderm and Nascent mesoderm, the second one in ExE endoderm and Visceral endoderm and the third one of Parietal endoderm and ExE ectoderm. The samples from Rostral neurectoderm and Surface ectoderm were discarded here as there was only one sample for each cell-type. In the clustering we obtained, the map is well reflected as the three main groups are retrieved, and the first and second groups are closer to each other than the third group. The kernel combination method yields a result similar in terms of groups, however the tree presents a different branching pattern. The kernel tree is presented in the supplementary figure of Additional file 2.

**Fig. 10:**
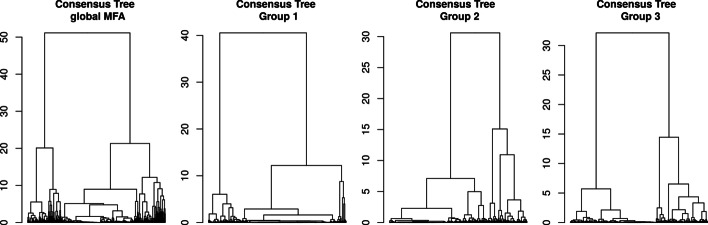
Consensus clusterings given by MFA for the single-cell data application. Hierarchical clustering obtained by using Euclidean distance and Ward’s aggregation criterion on the global MFA individuals (in this context, genes) coordinates and on the sub-groups MFA

Using the gene coordinates obtained with the MFA, we created a global consensus hierarchical clustering and three consensus trees corresponding to each group of identified cell-types. These trees are presented in Fig. 10 and the obtained groups of genes can be used for further functional analyses.

### Application to breast cancer data

The data used in this section are downloaded from the TCGA website using the curatedTCGAData [44]R-package.

#### Network integration

In this application, we will work at the protein level, this time building networks and considering them as the only source of data. The goal is once again to study how these objects can be grouped. We did not perform here further analyses of the individual coordinates, but it would also be possible.

Data are protein expression from 777 patients with breast cancer, divided into 4 subtypes: Basal-like ($n = 151$), HER2-enriched ($n = 85$), Luminal A ($n = 283$), Luminal B ($n = 258$). In this data set, $p = 173$ proteins were expressed in at least one sample of any subtype.

Using the limma R-package [45] to perform a differential analysis, we selected the 5 first proteins by order of adjusted p-value, for each contrast between subtypes, which provided 15 unique proteins. Networks associated with each subtype were inferred using glasso [46, 47] on centered data, and the Bayesian information criterion (BIC) [48] was used to select the adequate level of penalty, as implemented in the huge R-package [49]. All non-zero coefficients were set to 1 in the adjacency matrices. Using these networks as the set of objects we want to study, and the shortest path distance, we obtained new coordinates by MDS, that were then used in the MFA analysis. The hierarchical clustering based on the objects coordinates provided two groups, consisting in the Luminal A and B subtypes in one group and the HER2-enriched and Basal-like subtypes in the other.

**Fig. 11:**
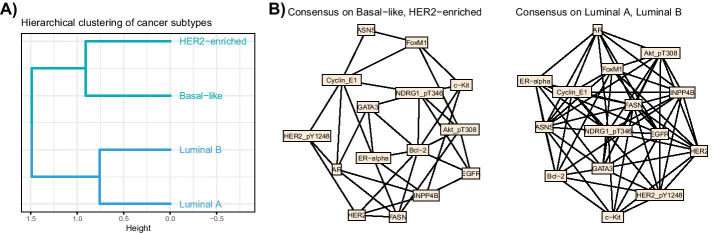
TCGA Breast cancer application. (**A**) Shows the hierarchical clustering of the breast cancer subtypes obtained with MFA group coordinates. (**B**) Shows the two consensus networks, made with a majority rule from the adjacency matrices from the subtype groups found in (**A**)

The clustering of the subtype networks, obtained on the MFA group coordinates, as well as for the consensus networks obtained by majority-rule are shown in Fig. 11. The results obtained for the kernel combination were exactly the same in this case in terms of network groups and therefore consensus networks.

#### Tree integration

In this part, we work on four omics data tables, which correspond to measures of methylation, mirna, protein and gene expression (RNA-seq data) for 113 patients with breast cancer. As before, the patients are classified into four subtypes: Basal($$n = 25$$), HER2-enriched ($$n = 19)$$, Luminal A ($$n = 35$$), Luminal B ($$n = 34$$). Features with null variance were removed prior to creating the trees. For the RNA-seq data, we also removed the genes having a mean count lower than 1 per sample, and transformed the data using $$x \mapsto \log _2(x+1)$$ transformation. These filters provided 222 proteins, 810 mirnas, 17,756 genes and 22,569 methylation sites.

**Fig. 12:**
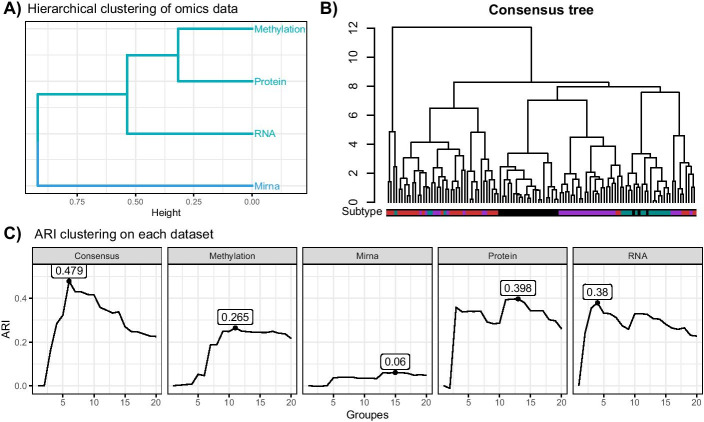
TCGA breast cancer multi-omics application. (**A**) Shows the hierarchical clustering of the breast cancer subtypes obtained with MFA group coordinates. (**B**) Shows the consensus tree obtained using the MFA individual coordinates. **C** Shows the adjusted rand index obtained for the individual trees and the consensus tree for 1 to 20 groups, with the maximum ARI highlighted

Results of the MDS and MFA combination, retaining 10 axes for the MDS and 5 axes for the MFA, are presented in Fig. 12. Figure 12C shows that the mirna table does not reflect the same information as the other tables, which is confirmed by the clustering of the tables presented in Fig. 12A. Indeed, the DynamicTreeCut procedure chose two groups: RNA-seq, protein and methylation data tables in the first one, and mirna alone in the second one.

We compared each individual tree and the consensus tree with the subtype classification using the Adjusted Rand Index (ARI). The mirna tree was found to have a lower ARI than all the other tables. The consensus tree made from the MFA axes is represented in Fig. 12B, with subtype indicated in the color bars. The consensus has a better ARI than all of the tables, demonstrating that the classification of the patients was improved by using this process. It was also robust to the low ARI value from mirna data table.

## Discussion

In this paper we proposed a procedure to compare multiple objects built on the same entities, with a focus on trees and networks, in order to define coherent groups of these kind of structures to be further integrated.

Because its computation only relies on a singular value decomposition (SVD), and since we may have recourse to a truncated version of SVD, the procedure is very fast and appropriate to analyze a great number of objects. Our procedure was applied to simulated data, both in the context of trees and networks. In both cases, three very different grouping information were generated. The method was able to retrieve these three different structures. Consensus trees and networks were then obtained based on the MFA results and were consistent with the simulated data for both the tree and network examples. We also analyzed two real data sets. A single-cell data set on mouse embryos was used to illustrate the performance of the methods on trees. Comparison with a clustering obtained in a previous study on these data [43] showed that the proposed methodology can integrate several trees while preserving the biological meaning of the data. A TCGA breast cancer data set was also used to illustrate the process on network data. It emphasized two groups of breast cancer subtypes that are consistent with the literature. It also allowed to create two consensus networks that highlight differences in the protein interactions in these two groups. In both simulations and real data application, the procedure was shown to be an efficient and useful tool for the user to identify groups of data that are relevant to integrate. This procedure was compared to a kernel integration method for each of the simulations, as well as the real data examples. The results were found to be quite similar. For further analyses following the creation of groups of tables we chose to use an unsupervised method (hierarchical clustering). It is possible to create the groups with other methods.

The kernel approach and the procedure we present here are closely related. They give similar results in terms of grouping objects together, but rely on a different manner to build a compromise (i.e. coordinates that are used to create a consensus), as seen in the tree integration simulation.

We studied here the integration of data of the same types (trees or networks), but our procedure can integrate them together, along with other types of representations. An interesting point to be further investigated would be the integration of additional information such as clinical data. This would indeed be possible thanks to the use of MFA that can deal with data of various types (continuous and categorical).

Any metric or transformation of the objects can be used as long as it yields a dissimilarity matrix usable in the MDS step. In this paper, we used binary adjacency matrices with shortest path distance for the networks, and cophenetic distances for the trees, and computed kernels derivated from these metrics. Any dissimilarity or distance measure, as well as adapted kernels, can be used in the process.

In the results presented here, we sticked to simple choices of methods to build the trees and networks, namely hierarchical agglomerative clustering and glasso. The use of different methods like self-organising maps [50] or bayesian hierarchical clustering [51] might lead to different results, especially as the MFA is an exploratory method. The unsupervised and exploratory aspects are particularly visible in the TCGA-tree application, where the consensus ARI was still quite low despite being an improvment compared to each individual tree. MFA is an unsupervised descriptive and exploratory method, and is therefore not dedicated to supervised analyses implicitly searching for differences among factors. However, such supervised analyses may be done in the framework of MFA using for example the multiblock redundancy analysis [52], extending the redundancy analysis, which is a supervised version of PCA [53] to a multiblock context.

The process was applied here on real-life datasets where the separation between objects was clear, and where there was an informative signal in the omics datasets. It might be more difficult in the case of multifactorial diseases with less information in the multi-omics measurements.

The rationale of the method lies in the possibility of visualizing the data in a Euclidean space, whatever their original form (network, dendrogram, etc.) We stayed in a Euclidean setting in our simulations and applications. In some cases, the use of Euclidean representation might not be suitable or wanted by the user as it might distort the information. In this particular setting, the use of the kernel combination is needed, but will not allow representation of the results as factorial maps and will therefore be less interpretable.

We have illustrated our method with the shortest path distance and metric MDS, but this is not a requisite, and our method can be easily extended to any combination of distance and dimensional scaling (metric or not metric), provided it leads to such a Euclidean representation.

## Conclusion

In this paper we proposed a procedure to compare multiple objects built on the same entities, with a focus on trees and networks, in order to define coherent groups of these kind of structures to be further integrated.

The procedure relies on two well-known methodologies, namely multidimensional scaling (MDS) and Multiple Factor Analysis (MFA), that offer a unified framework to analyze both tree or network structures. The proposed approach provides tools to compare the structures and to easily obtain consensus trees or networks.

The use of MFA allows the users to access a great number of libraries to help visualize the results, as well as to perform further analyses on individual coordinates.

## Supplementary Information

**Additional file 1.** Results for the simulation study on hierarchical clustering data. Individual coordinates on the four first factorial axes from the MFA, colored according to each of the simulated classification.

**Additional file 2.** Visualization of groups given by kernel combination for the single-cell data application. A) Dendrogram of the cell-types obtained on the *C*-coefficient matrix, using complete-linkage on the transformed similarities. Clusters were chosen using DynamicTreeCut and colored accordingly. B) Heatmapof the $C$-coefficient between tables. These similarities were transformed into dissimilarities and used to create the hierarchical clustering in panel A. The black grid shows the clusters as found in the dendrogram of panel A.

## Data Availability

The simulated datasets analysed during the current study are available at https://github.com/AudreH/intTreeNet. TCGA BRCA methylation, mirna, protein and gene expression datasets were downloaded using the curatedTCGAData R-package and can alternatively be accessed through their portal at https://portal.gdc.cancer.gov/. The single-cell datasets analysed during the current study are available using the R-package *mouseGastrulationData* on Bioconductor https://bioconductor.org/packages/release/data/experiment/html/MouseGastrulationData.html.

## Abbreviations

ARI: Adjusted Rand Index
BIC: Bayesian information criterion
MDS: Multidimensional scaling
MFA: Multiple factor analysis
PCA: Principal component analysis
SVD: Singular value decomposition
TCGA: The Cancer Genome Atlas
