# Supplementary figures and images for: A unified framework for the integration of multiple hierarchical clusterings or networks from multi-source data
Source: BMC Bioinformatics. 2021 Aug 4;22:392. doi: 10.1186/s12859-021-04303-4 (PMC8336092; doi:10.1186/s12859-021-04303-4)

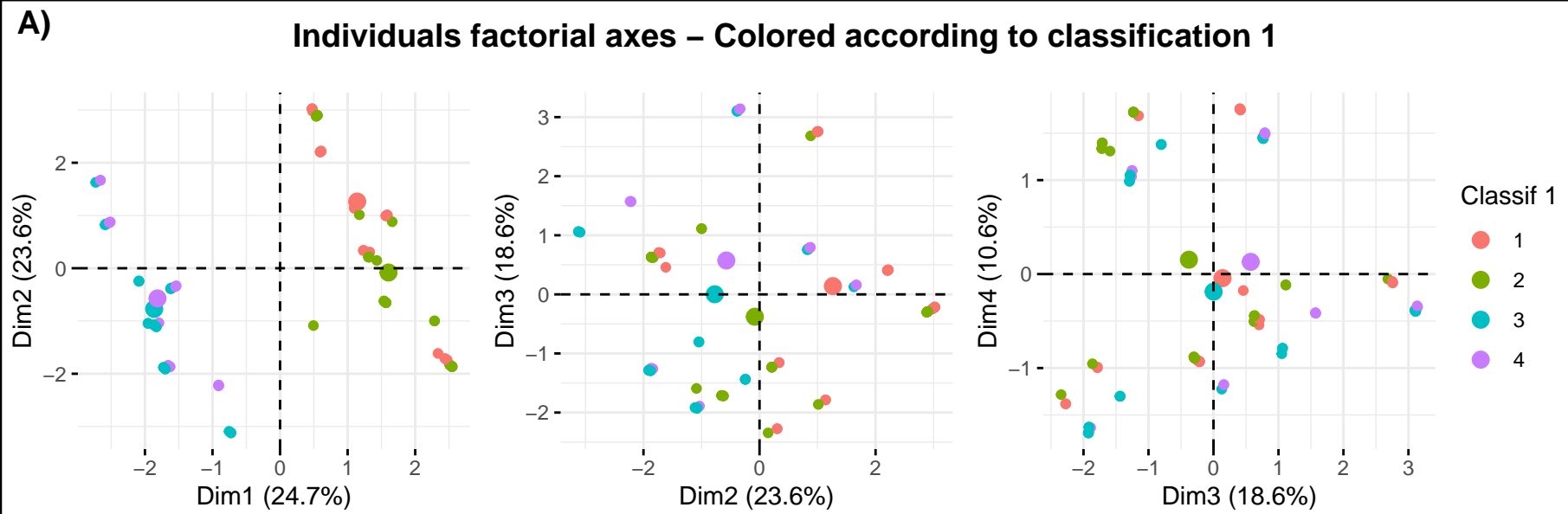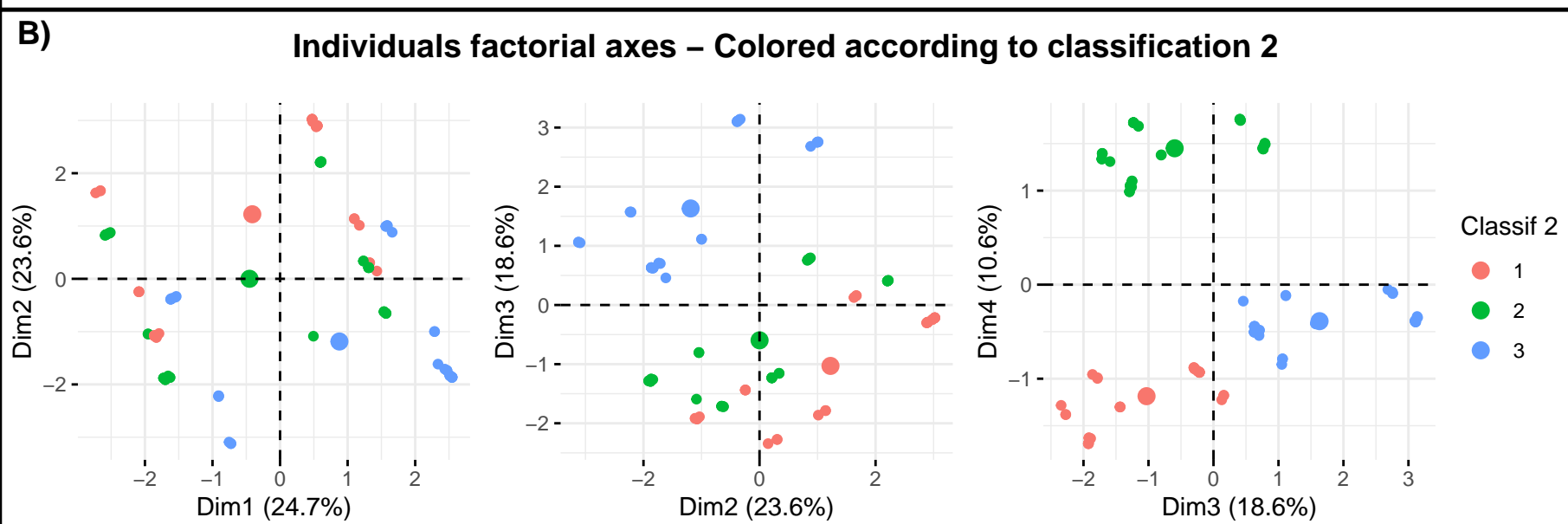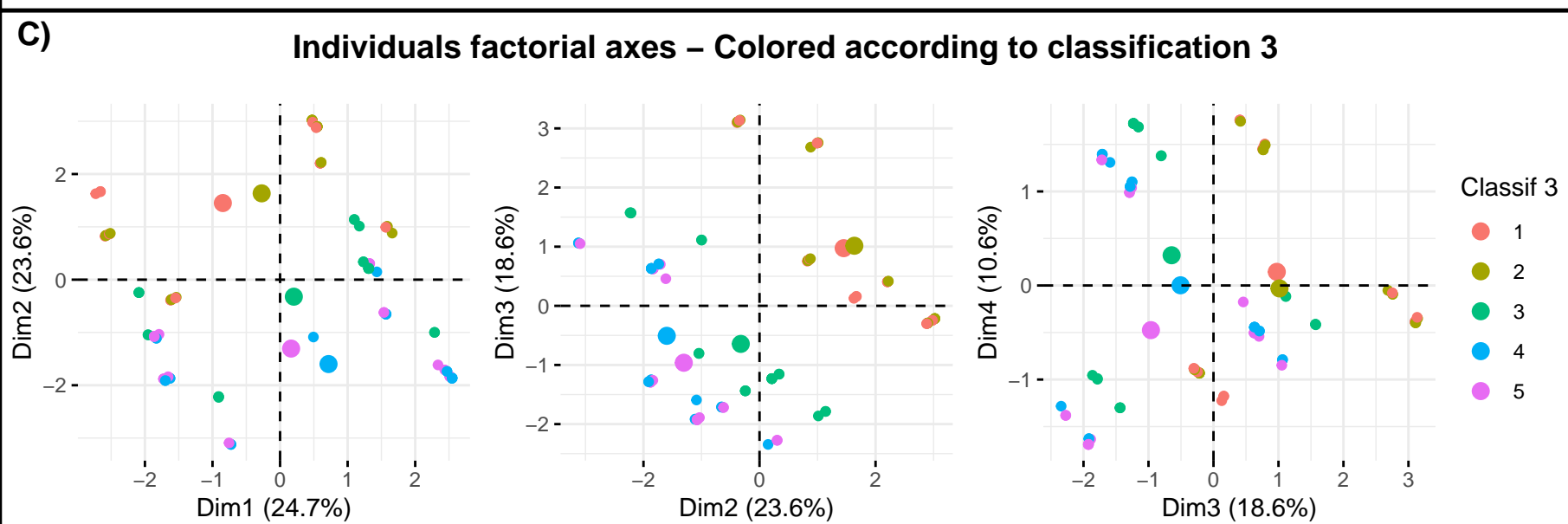

Supplement: Supplementary file 1 — Additional file 1. Results for the simulation study on hierarchical clustering data. Individual coordinates on the four first factorial axes from the MFA, colored according to each of the simulated classification. [file 12859_2021_4303_MOESM1_ESM.pdf]

A) Hierarchical Clustering of cell types

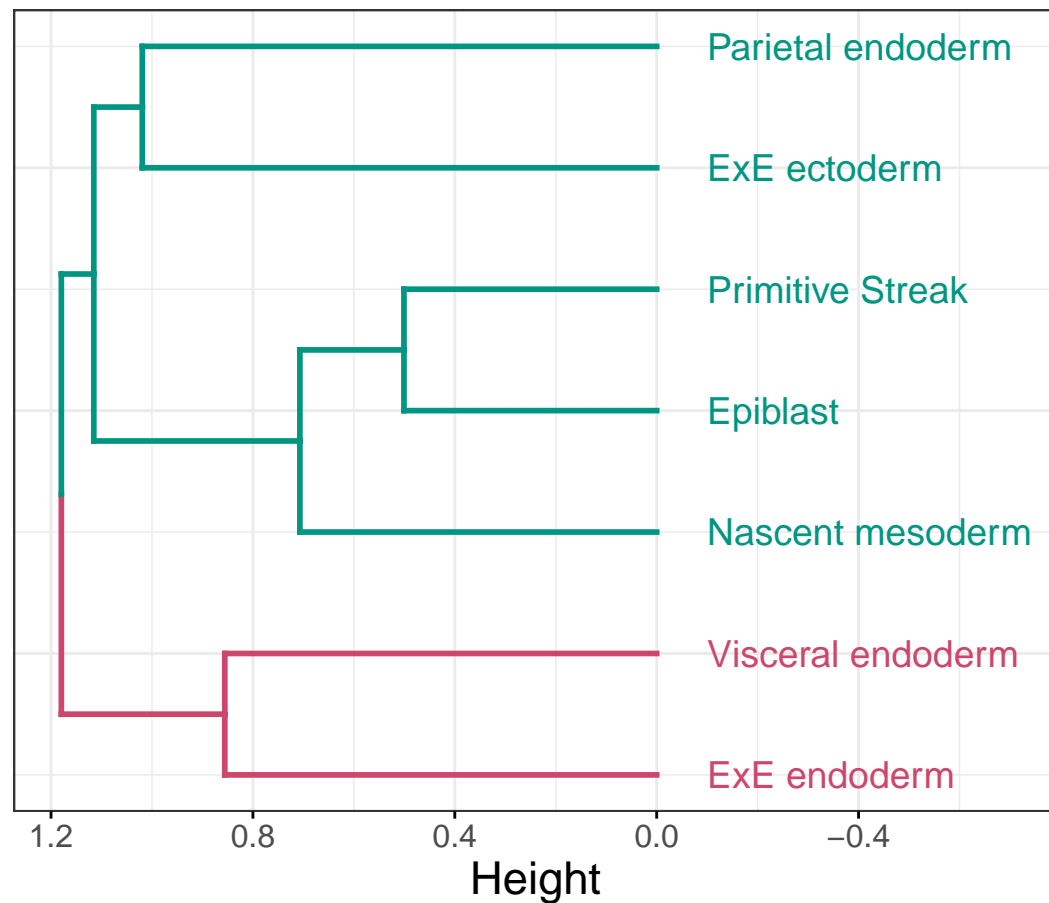

B)

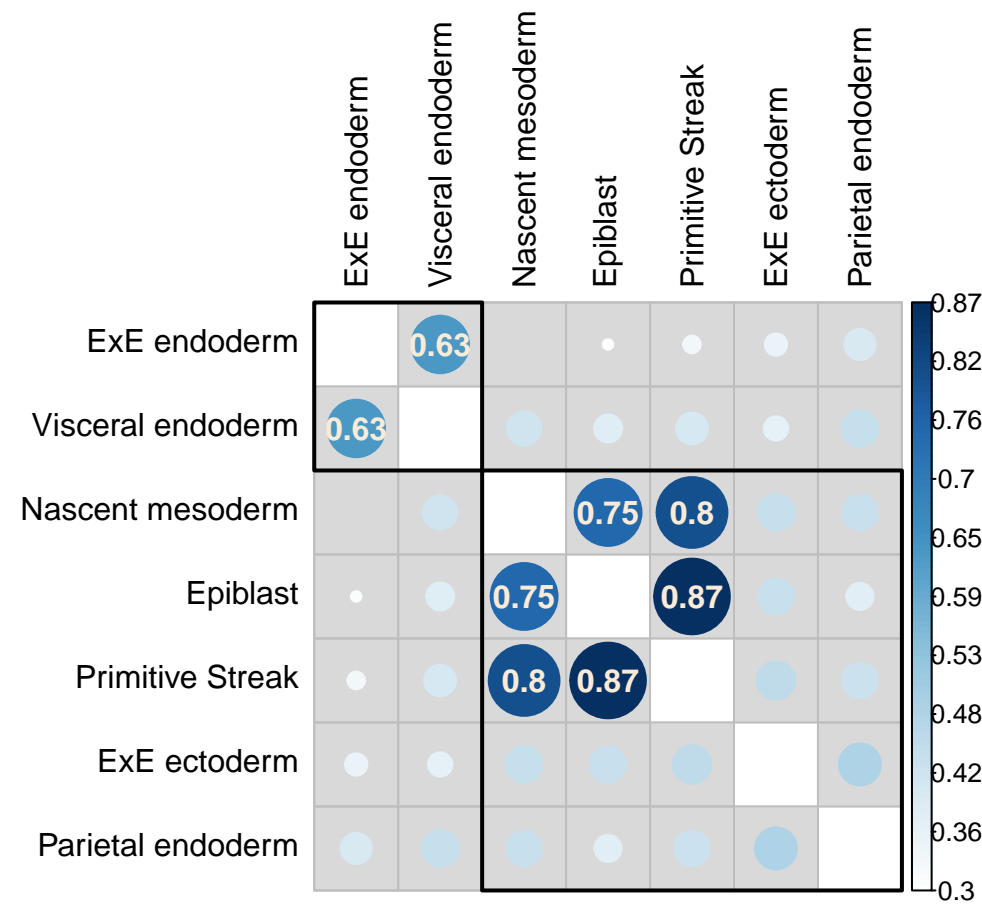

Supplement: Supplementary file 2 — Additional file 2. Visualization of groups given by kernel combination for the single-cell data application. A) Dendrogram of the cell-types obtained on the C-coefficient matrix, using complete-linkage on the transformed similarities. Clusters were chosen using DynamicTreeCut and colored accordingly. B) Heatmapof the $C$-coefficient between tables. These similarities were transformed into dissimilarities and used to create the hierarchical clustering in panel A. The black grid shows the clusters as found in the dendrogram of panel A. [file 12859_2021_4303_MOESM2_ESM.pdf]
